# Magnetic field sensor based on magnetoplasmonic crystal

**DOI:** 10.1038/s41598-020-63535-1

**Published:** 2020-04-28

**Authors:** Victor K. Belyaev, Valeria V. Rodionova, Andrey A. Grunin, Mitsuteru Inoue, Andrey A. Fedyanin

**Affiliations:** 1 Institute of Physics, Mathematics and Informational Technologies, Immanuel Kant Baltic Federal University, Kaliningrad, 236041 Russia; 2 Faculty of Physics, Lomonosov Moscow State University, Moscow, 119991 Russia; 3 Toyohashi University of Technology, Toyohashi, 441-8580 Japan

**Keywords:** Sensors, Optical sensors

## Abstract

Here we report on designing a magnetic field sensor based on magnetoplasmonic crystal made of noble and ferromagnetic metals deposited on one-dimensional subwavelength grating. The experimental data demonstrate resonant transverse magneto-optical Kerr effect (TMOKE) at a narrow spectral region of 50 nm corresponding to the surface plasmon-polaritons excitation and maximum modulation of the reflected light intensity of 4.5% in a modulating magnetic field with the magnitude of 16 Oe. Dependences of TMOKE on external alternating current (AC) and direct current (DC) magnetic field demonstrate that it is a possibility to use the magnetoplasmonic crystal as a high-sensitive sensing probe. The achieved sensitivity to DC magnetic field is up to 10^−6^ Oe at local area of 1 mm^2^.

## Introduction

Development of magnetic field sensors primarily works on improving the sensor’s design and its characteristics - sensitivity, resolution, locality and reliability as well as on extending the sensor’s operating temperature range or conditions of applicability (like harsh environments)^1–4^. One of the cutting-edge topics today is using the magnetic field sensors in medical applications such as magnetocardiography^5,6^ and magnetotomography^7^, which require precise measurements of magnetic field with the magnitude reaching 1 μOe. Nowadays the most reliable techniques are based on SQUIDs^5,8^, induction coil sensors^9,10^ and Hall-effect sensors^11,12^. These methods have several limitations connected with the decrease of sensitivity in small volumes^13,14^, the low temperatures requirement or moving the sensor probe for changing the sensing area. The alternative approach utilizes magneto-optical effects in transmission or reflection configurations, which allow one to preserve sensitivity at a local point and to scan the certain volume without moving the sensing element^15–17^. In this case the locality depends only on the optical beam size and the penetration depth of optical radiation in the medium, while the sensitivity is proportional to magnetization of the medium and the scanning volume can be changed by moving the optical spot.

One of the possible ways to increase the sensitivity of magneto-optical sensors is to get surface plasmon-polaritons (SPPs) excited on the metal-dielectric interface. SPPs induce the resonant magneto-optical effects appearing^18–22^. They increase the polarization plane rotation or modulation of the reflected (transmitted) light intensity utilizing the nonreciprocity of magneto-optical effects. The surface plasmons, being the electromagnetic excitations at the metal surface consisting of polaritons and electronic gas oscillations, require fulfilling the phase-matching conditions applying various experimental schemes, such as Kretchman, Otto and grating configurations^23^. Magneto-optical effects can be enhanced by SPPs in magnetoplasmonic crystals fabricated of noble metal and magnetic layers with one- or two-dimensional subwavelength grating^20,24–27^. There are several examples of using the magnetoplasmonic excitations in magneto-optical sensors of biomolecules^15,28^, chemical solutions^29,30^ and gases^31^ through detecting the ultralow refractive index changes^32^. Another promising direction is using the localized surface plasmons excited in metallic and hybrid nanoantennas^17,33,34^.

In this Letter, we show how the magnetoplasmonic crystal can operate as a highly sensitive local sensor of DC magnetic field. The use of controllable AC magnetic field allows one to choose the certain region at the magneto-optical response curve with the strongest dependence of the signal on the magnetic field magnitude. The sensor measures the DC magnetic field component parallel to the AC magnetic field while the shift of optical beam reveals the magnetic field distribution in the desired volume. The enhanced TMOKE, achieved due to excitation of SPPs, allows us to detect the magnetic field with sensitivity reaching 10^−6^ Oe.

## Results and Discussions

### Sample characteristics and geometry

Magnetoplasmonic crystals were fabricated by ion-beam deposition of noble (silver) and ferromagnetic (iron) metal layers onto the surface with quasi-sinusoidal subwavelength polymeric grating. Before the fabrication the chamber was vacuumed down to 8 · 10^−7^ Torr. During the fabrication process the argon flow of 6 ccm and ion source MPC-3000HC with working current of 30 mA and voltage of 1000 V were used. All the substrates were rotating during the fabrication process to avoid the shadowing effect. The period and profile height of the grating were equal to 320 and 20 nm, respectively^35,36^. Surface of magnetoplasmonic crystals was passivated by a thin transparent layer of dielectric (silica nitride) to prevent oxidation of the iron layer. Thicknesses of functional layers were varied to estimate the contributions of magnetic and plasmonic properties into the enhancement of TMOKE and sensitivity of DC magnetic field sensor based on MPlCs. The samples with thickness of iron layer above 50 nm can be considered as pure ferromagnetic gratings where magnetic contribution is dominant, while in the other samples the contribution of silver layer starts to play an important role in forming the magneto-optical response due to the increase of SPPs free mean path and extending the interaction of light with ferromagnetic material. The thickness parameters of functional layers of magnetoplasmonic crystals are listed in Table 1.

**Table 1:** Composition of magnetoplasmonic crystals.

Sample number	Layer material (thicknesses in nm)
Sample 1	Ag(100)/Fe(100)/Si_3_N_4_(20)
Sample 2	Ag(100)/Fe(50)/Si_3_N_4_(20)
Sample 3	Ag(100)/Fe(20)/Si_3_N_4_(20)
Sample 4	Ag(100)/Fe(5)/Si_3_N_4_(20)

The surface profile and deposited layer thickness were examined by atomic force microscope (AFM) and scanning electron microscope (SEM), the magnetic properties were measured by vibrating sample magnetometer (VSM). Optical and magneto-optical properties were studied by a setup made up of the halogen lamp with a monochromator serving as a light source, Glan-Taylor prism as polarizer, a photomultiplier tube (model H10722-20 by Hamamatsu) with a lock-in amplifier as a detector accompanied by an optical chopper that controls the frequency of an optomechanical modulation or a system of electromagnets which allowed us to control the magnitudes of AC and DC magnetic field.

**Figure 1:**
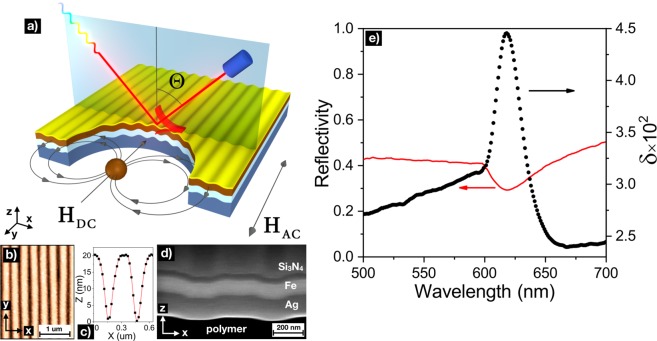
Panel (a) Schematic view of the sensing element - the magnetoplasmonic crystal with surface plasmon excited on silver-iron bilayer, *H*_*AC*_ and *H*_*DC*_ represent external control AC magnetic field and DC stray fields of the probe object, respectively. Panels (b-d) The AFM image of the surface, extracted profile and the SEM cross-section image of the Sample 1, respectively. Panel (e) shows reflectivity and TMOKE spectra for the Sample 1.

The setup schematics and the sample’s design were optimal to be measured in TMOKE geometry according two reasons: (i) the magnetic field was applied in-plane along the easy magnetization axis, and gave the highest ratio of the magnetic moment modulation in fields with magnitude below 50 Oe^36^; (ii) the useful signal (relative magnitude of intensity changes on photodetector) on in magneto-optic measuring schemes for TMOKE is commonly more in comparison with LMOKE. For maximisation of the TMOKE signal measurements were carried out in the p-polarized light with the incidence angle fixed to Θ = 68°^35^, frequencies of optomechanical and AC magnetic field, *H*_*AC*_, modulations were chosen to be 233 and 317 Hz, respectively. The illuminated spot sizes were 12 mm^2^ and 1 mm^2^. Figure 1 shows the schematic view of the sensing element, the spatial profile of magnetoplasmonic crystal obtained by AFM and the SEM cross-section imaging.

### Experimental demonstration

The TMOKE value is defined as *δ* = (*R*_+*H*_ + *R*_−*H*_)/*R*_0_, where *R*_0_ is the reflection amplitude without magnetic field, which was detected with optomechanical modulation of the incident light, *R*_+*H*_ and *R*_−*H*_ denote the field dependent reflection amplitudes. Measurements of spectral dependencies of reflectivity and TMOKE were carried out in saturation AC magnetic field of 50 Oe. Reflection and TMOKE spectra for Sample 1 are shown in Fig. 1d.

The minimum of the specular reflectivity and the maximum of the TMOKE signal are clearly observed at the resonant wavelength of 618 nm and related to strong coupling of plasmon oscillations and the light diffracted into the -1^*st*^ order^23^. The excited SPPs tightly localize the electric field of the incident electromagnetic wave at the Fe/Si_3_N_4_ interface that leads to efficient light-matter interaction and results into the resonant enhancement of TMOKE.

**Figure 2:**
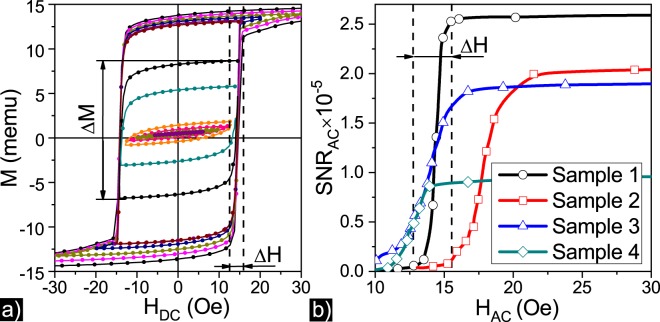
Panel (a) Set of minor hysteresis loops obtained by demagnetizing the Sample 1 using VSM. Panel (b)Dependences of *SNR*_*AC*_ for all samples.

**Figure 3:**
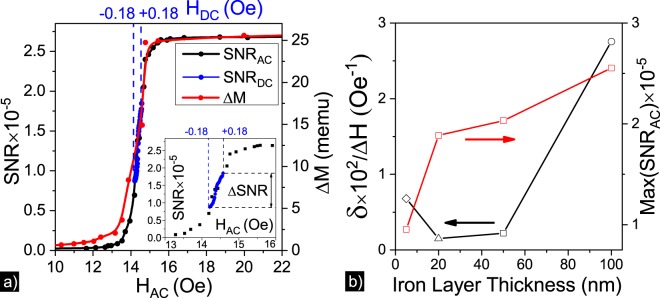
Panel (a) shows the magnetic field dependences of SNR and Δ*M* for the Sample 1. Blue dashed lines show DC magnetic field range. Inset zooms the central part of the SNR dependence. Panel (b) shows dependences of *δ*/Δ*H* and *Max*(*SNR*_*AC*_) values on iron layer thickness.

Figure 2a shows the set of minor hysteresis loops measured by VSM from the saturation magnetic field of *H*_*sat*_ = 50 Oe: the field magnitude was gradually decreased by a small step value of *H*_*step*_ for measuring the hysteresis loop in magnetic field down to *H*_*n*_ = *H*_*sat*_ − *n *·* H*_*step*_, where *n* is a step number. By this way the sample was demagnetized and values of $\Delta M({H}_{n})=M(\,+\,{H}_{n})-M(\,-\,{H}_{n})$ were obtained (Fig. 3a, solid red curve). The Δ*H* value shown by dashed lines corresponds to the region of rapidly decreasing Δ*M*(*H*) and denotes the field region of hysteresis loop collapse.

The noise of the sensor prototype is measured at the resonant wavelength and saturation magnetic field as the time dependence of PMT voltage output for 500 points with 3 seconds per point. Then, the standard deviation $\sigma =\sqrt{{({x}_{N}-\bar{x})}^{2}/(N-1)}$, where *N* is a number of acquisition points, is used to calculate the signal-to-noise ratio $SN{R}_{AC}=({R}_{+H}-{R}_{-H})/\sigma$, where (*R*_+*H*_ − *R*_−*H*_) value were accumulated for 3 seconds at a given magnetic field and sensitivity Δ*SNR*/Δ*H*, where Δ*SNR* is the difference of maximum and minimum *SNR* values in selected Δ*H* range.

Figure 2b shows the dependences of the signal-to-noise ratio *SNR*_*AC*_ on AC magnetic field for all samples. The *SNR*_*AC*_ dependences have a step-like behaviour: in AC magnetic field with an amplitude of the saturation field, *SNR*_*AC*_ has the maximum value and starts to decrease to zero with decrease of the magnetic field. The width of the step for the Sample 1 is Δ*H* = 2.8 Oe and shown by the dashed lines which corresponds the field region of hysteresis loop collapse shown in Fig. 2a.

The *SNR*(*H*_*DC*_) dependence was obtained by (i) setting of *H*_*AC*_ value to the maximum of the derivative $\partial SN{R}_{AC}/\partial {H}_{AC}$ that allows one to get the point in the center of the observed slope of *SNR*(*H*_*AC*_) dependence and (ii) application of additional magnetic field *H*_*DC*_ with the magnitude of ±0.18 Oe. This way *SNR*(*H*_*DC*_) was measured for all samples and compared with the relative changes of iron layer magnetic moment Δ*M* obtained by demagnetizing the sample using VSM. The shape of magneto-optical response dependence on magnetic field correlates with the relative changes in magnetic moment of iron layer which can be written as $\delta =({R}_{+H}+{R}_{-H})/{R}_{0}\sim \Delta M={M}_{+H}-{M}_{-H}$. Typical dependences of *SNR*(*H*_*AC*_), *SNR*(*H*_*DC*_) and Δ*M*(*H*) are presented in Fig. 3a.

*SNR*_*AC*_ and *SNR*_*DC*_ dependences show that the magneto-optical response depends on a sum of magnitudes of AC and DC magnetic fields affecting the magnetoplasmonic crystal in the direction perpendicular to the plane of light incidence and proportional to a magnetic moment of ferromagnetic layer. It is possible to use the *SNR*_*DC*_ dependence as a calibration curve for estimating the reliable and precise correlation between the field dependent magneto-optical response and the external field magnitude.

Two functions are considered to reveal the dependence of magnetic field sensors sensitivity on the iron layer thickness in magnetoplasmonic crystals. The first one, *δ*/Δ*H*, is shown in Fig. 3b and depends both on the maximum modulation of optical reflectance by magnetic field at the wavelengths corresponding to excitation of SPPs and on the width of the step in *SNR*_*AC*_ dependence. The second dependence, *Max*(*SNR*_*AC*_), describes the dependence of *SNR*_*AC*_ at saturation magnetic field on the thickness of the iron layer in magnetoplasmonic crystals. Variation of the iron layer thickness allows one to tune the sensitivity by changing optical and magnetic properties of magnetoplasmonic crystals. Magnetic moment and optical losses monotonously increase with the iron layer thickness, while the shape of the *δ*/Δ*H* dependence is mostly determined by non-monotonic changes of the coercive force and Δ*H* value^36^. The shape of Max(*SNR*_*AC*_) strongly depends on the iron layer magnetization and monotonously increases with the growing iron layer thickness. The sensitivities of DC magnetic field sensor prototypes based on magnetoplasmonic crystals are estimated to be 3.7 · 10^−6^, 3.2 · 10^−5^, 3.4 · 10^−5^ and 3.8 · 10^−5^ Oe at a room temperature for iron layer thickness of 100, 50, 20 and 5 nm, respectively. Thus, it is shown that sensing capabilities are stronger correlated with the value of magnetic moment, than with the plasmonic properties and value of optical losses. The highest sensitivity is achieved for the sample with the iron layer thickness of 100 nm.

Further increase of the TMOKE value is achieved by optimizing the illuminated spot size. For Sample 1 the *SNR*_*AC*_ value at saturation magnetic field is changed from 2.7 · 10^5^ to 3.2 · 10^5^ with decreasing the spot size from 12 mm^2^ to 1 mm^2^ due to the difference in magnetization processes: using a small region in the center of magnetoplasmonic crystal allows one to increase the steepness of the magnetization curve by neglecting the edge effects which lead to domain nucleation with opposite magnetization direction in lower magnetic field. With the decrease of the spot size the value of sensitivity changes from 3.7 · 10^−6^ to 3.1 · 10^−6^. The minimal optical spot size to use the magnetoplasmonic crystal as a magnetic field sensor is determined by the following parameters: diffraction limit, wavelength of SPPs excitation and fulfilling the diffraction conditions and is estimated to be as small as 5 μm^2^. The theoretical limit of sensitivity of 10^−7^ Oe is estimated as a sum of four noise sources, namely, of thermal ${i}_{th}=\sqrt{4kTR\Delta f}$, flicker ${i}_{fl}=1/{f}^{\gamma }$, shot ${i}_{sh}=\sqrt{(2{q}_{e}I\Delta f)}$, and avalanche ${i}_{av}=\sqrt{(2{q}_{e}I/2\pi )}$ noises and did not exceed the value of 6 · 10^−9^ that was by two orders smaller than the measured noise value. Table 2 compares the sensitivity and locality of various magnetic field sensors and reveals the advantages of the designed sensing element. The sensor based on magnetoplasmonic crystal as a probe provides high sensitivity at small spot size which makes it sufficient and promising for biomedical applications and allows one to scan the surface area without moving the probe element.

**Table 2:** Comparison of sensors in terms of the probe sensitivity and locality.

Magnetic field sensor type	Hall effect^12,37,38^	Magneto-modulation^9,13,39,40^	Magneto-optical^41,42^	Magnons^16^	Magnetoplasmonic	
					Prototype	Theory
Sensitivity	10^−6^ Oe	10^−7^ Oe	10^−2^ Oe	10^−9^ Oe	3 · 10^−6^ Oe	10^−7^ Oe
Probe Size	200 nm^2^	1 mm^2^	5 μm^2^	4 · 9 cm^2^	1 mm^2^	5 μm^2^

## Conclusions

Summarizing, here we demonstrate DC magnetic field sensors based on magnetoplasmonic crystals made of noble and ferromagnetic metals deposited on one-dimensional subwavelength grating utilizing TMOKE enhanced by excitation of SPPs. The correlation between magneto-optical and magnetic properties reveals the possibility to tune the sensitivity of the sensor by changing the ferromagnetic layer thickness. The sensitivity of the sensor prototype based on the magnetoplasmonic crystal was found to be 3 · 10^−6^ Oe with the spot size of 1 mm^2^ and can be further improved by optimizing the sensing element and the sensor’s setup overall design.

## References

[1] Tumanski S (2013). Modern magnetic field sensors - a review. Prz. Elektrotech..

[2] Zhai J, Xing Z, Dong S, Li J, Viehland D (2006). Detection of pico-Tesla magnetic fields using magneto-electric sensors at room temperature. Appl. Phys. Lett..

[3] Stutzke NA, Russek SE, Pappas DP, Tondra M (2005). Low-frequency noise measurements on commercial magnetoresistive sensors. J. Appl. Phys..

[4] Budker D, Romalis M (2007). Optical magnetometry. Nat. Physics.

[5] Tsukada K (1995). Multichannel SQUID system detecting tangential components of the cardiac magnetic field. Rev. Sci. Instrum..

[6] Koch H (2004). Recent advances in magnetocardiography. J. Electrocardiol..

[7] Marmugi L, Renzoni F (2016). Optical magnetic induction tomography of the heart. Sci. Rep..

[8] Drung D (2007). Highly sensitive and easy-to-use SQUID sensors. IEEE Trans. Appl. Supercond..

[9] Tumanski S (2007). Induction coil sensors: A review. Meas. Sci. Technol..

[10] Deans C, Marmugi L, Hussain S, Renzoni F (2016). Electromagnetic induction imaging with a radio-frequency atomic magnetometer. Appl. Phys. Lett..

[11] Michele Ldi (2011). Detection and susceptibility measurements of a single Dynal bead. J. Appl. Phys..

[12] Sandhu A, Okamoto A, Shibasaki I, Oral A (2004). Nano and micro Hall-effect sensors for room-temperature scanning hall probe microscopy. Microelectron. Eng..

[13] Ripka P, Janosek M (2010). Advances in magnetic field sensors. IEEE Sens. J..

[14] Grigorashvili Yu ELP, Volik NN (2006). Magnetomodulation sensor of a weak magnetic field based on HTS (Bi, Pb)2Sr2Ca2Cu3Ox ceramics. Physica C.

[15] Sepulveda B, Calle A, Lechuga LM, Armelles G (2006). Highly sensitive detection of biomolecules with the magneto-optic surface-plasmon-resonance sensor. Opt. Lett..

[16] Inoue M (2011). Investigating the use of magnonic crystals as extremely sensitive magnetic field sensors at room temperature. Appl. Phys. Lett..

[17] Maccaferri N (2015). Ultrasensitive and label-free molecular-level detection enabled by light phase control in magnetoplasmonic nanoantennas. Nat. Commun..

[18] Krinchik GS, Chepurova EE, Kraeva TI (1984). Excitation of magnetized-plasma surface-waves in nickel. JETP Lett..

[19] Clavero C, Yang K, Skuza JR, Lukaszew RA (2010). Magnetic field modulation of intense surface plasmon polaritons. Opt. Express..

[20] Grunin AA, Zhdanov AG, Ezhov AA, Ganshina EA, Fedyanin AA (2010). Surface-plasmon-induced enhancement of magneto-optical Kerr effect in all-nickel subwavelength nanogratings. Appl. Phys. Lett..

[21] Chetvertukhin AV (2012). Magneto-optical Kerr effect enhancement at the Wood’s anomaly in magnetoplasmonic crystals. J. Magn. Magn. Mater..

[22] Belotelov VI (2013). Plasmon-mediated magneto-optical transparency. Nat. Commun..

[23] Zayats A, Smolyaninov I (2003). Near-field photonics: surface plasmon polaritons and localized surface plasmons. J. Opt. A: Pure Appl. Opt..

[24] Belotelov VI (2011). Enhanced magneto-optical effects in magnetoplasmonic crystals. Nat. Nanotechnol..

[25] Shcherbakov MR, Vabishchevich PP, Frolov AY, Dolgova. TV, Fedyanin AA (2014). Femtosecond intrapulse evolution of the magneto-optic Kerr effect in magnetoplasmonic crystals. Phys. Rev. B.

[26] Grunin AA, Sapoletova NA, Napolskii KS, Eliseev AA, Fedyanin AA (2012). Magnetoplasmonic nanostructures based on nickel inverse opal slabs. J. Appl. Phys..

[27] Belyaev VK, Grunin AA, Fedyanin AA, Rodionova VV (2015). Magnetic and magneto-optical properties of magnetoplasmonic crystals. Solid State Phenom..

[28] David S (2015). Magneto-plasmonic biosensor with enhanced analytical response and stability. Biosens. Bioelectron..

[29] Chou KH (2014). Application of strong transverse magneto-optical Kerr effect on high sensitive surface plasmon grating sensors. Opt. Express.

[30] Manera MG, Ferreiro-Vila E, Garcia-Martin JM, Garcia-Martin A, Rella R (2014). Enhanced antibody recognition with a magneto-optic surface plasmon resonance (MO-SPR) sensor. Biosens. Bioelectron..

[31] Ignatyeva DO (2016). Magneto-optical plasmonic heterostructure with ultranarrow resonance for sensing applications. Sci. Rep..

[32] Grunin AA, Mukha IR, Chetvertukhin AV, Fedyanin AA (2016). Refractive index sensor based on magnetoplasmonic crystals. J. Magn. Magn. Mater..

[33] Jeong HJ, Kim D, Song JH, Jeong KY, Seo MK (2016). Resonant magneto-optic Kerr effects of a single Ni nanorod in the Mie scattering regime. Opt. Express.

[34] Barsukova MG (2017). Magneto-optical response enhanced by Mie resonances in nanoantennas. ACS Photonics.

[35] Grunin AA, Chetvertukhin AV, Dolgova TV, Ezhov AA, Fedyanin AA (2013). Magnetoplasmonic crystals based on commercial digital discs. J. Appl. Phys..

[36] Belyaev VK, Kozlov AG, Ognev AV, Samardak AS, Rodionova VV (2019). Magnetic properties and geometry-driven magnetic anisotropy of magnetoplasmonic crystals. J. Magn. Magn. Mater..

[37] Boero GG (2005). Submicrometer Hall devices fabricated by focused electron-beam-induced deposition. Appl. Phys. Lett..

[38] Gabureac MS, Bernau L, Boero G, Utke I (2013). Single superparamagnetic bead detection and direct tracing of bead position using novel nanocomposite nano-hall sensors. IEEE Trans. Nanotechnol..

[39] Poliakov SV, Reznikov BI, Shchennikov AV, Kopytenko EA, Samsonov V (2017). The range of induction-coil magnetic field sensors for geophysical explorations. Seism. Instr..

[40] Ripka P, Pribil M, Petrucha V, Grim V, Draxler K (2016). A fluxgate current sensor with an amphitheater busbar. IEEE Trans. Magn..

[41] Koschny M, Lindner M (2012). Magneto-optical sensors accurately analyze magnetic field distribution of magnetic materials. Adv. Mat. and Proc..

[42] Valente J (2015). A magneto-electro-optical effect in a plasmonic nanowire material. Nat. Commun..

